# Genetic algorithm for obtaining potential energy curve of diatomic molecules based on dispersed fluorescence spectra

**DOI:** 10.1038/s41598-023-44488-7

**Published:** 2023-10-13

**Authors:** Tomasz Urbańczyk, Jarosław Koperski, Gabriel Kaszewski, Mikołaj Synak, Jakub Swenda, Marek Krośnicki

**Affiliations:** 1 Smoluchowski Intitute of Physics, Jagiellonian University in Krakow, Łojasiewicza 11, 30-348 Kraków, Poland; 2 Institute of Theoretical Physics and Astrophysics, Faculty of Mathematics, Physics and Informatics, University of Gdańsk, Wita Stwosza 57, 80-308 Gdańsk, Poland

**Keywords:** Chemical physics, Atomic and molecular physics

## Abstract

The method for reconstruction of an adiabatic potential energy curve from experimental dispersed fluorescence spectra has been developed. The novelty of the method relies on a unique approach of simultaneous use of $bound \rightarrow bound$ and $bound \rightarrow free$ parts of the spectrum. The method is based on the Genetic Algorithm (GA) procedure and determines potential energy curve integrally, below and above the dissociation energy limit. The method was tested on the artificially generated reference spectrum as well as experimental spectrum of $G0_u^+(\upsilon ^{\prime }=39) \rightarrow X0_g^+$ transition in Hg$_2$. The tests show very good accuracy of simulation based on GA results with artificially generated reference spectrum as well as with the experimental one.

## Introduction

One of the main goals of measurements of spectra of diatomic van der Waals molecules is to determine the molecular potentials of electronic states involved in the studied transitions. The determination of molecular potential (potential energy curve, PEC) on the basis of the experimental spectrum is an example of the problem that is difficult to reverse. Having potentials of electronic states that are involved in the transition, it is very easy to determine the spectrum of the transition by solving the corresponding Schrödinger equation. This can be performed using programs such as LEVEL^1^ or DUO^2^ (for *bound-bound* spectra) or BCONT^3^ (for *bound-free* spectra).

On the other hand, obtaining PEC based on experimental spectrum usually is more complicated. In simplest cases, where the analyzed potential can be represented by a Morse function, the Birge-Sponer (B-S) method can be used to determine the potential parameters. In more complicated cases, the PEC below the dissociation limit can be obtained using Rydberg-Klein-Rees (RKR) method^4^ or inverse perturbation approach (IPA) method^5^. Recently, we also show that the approach based on machine learning, which uses the neural network, can be employed to independently determine PEC below^6^ and above^7^ the dissociation limit. However, to the best of our knowledge a method of obtaining the whole PEC based on both $bound-bound$ and $bound-free$ spectra has not been presented so far. In this paper, we present the method that employs Genetic Algorithm (GA) which can be used to obtain parameters of analytical representation of PEC, which is valid below and above the dissociation limit. The GA is an optimisation technique which is inspired by the process of evolution. GAs are used in various fields of knowledge such as economics (*e.g.* for creating stock price forecasting model^8^), biology (*e.g.* for alignment of nucleic and amino acid sequences^9^) or climatology (*e.g.* for modeling global temperature changes^10^). In our previous work^11^ we have shown, that the GA can be also used to determine the shape of PEC of diatomic molecules below the dissociation limit based on $bound \leftarrow bound$ LIF excitation spectra with resolved ro-vibrational energy structure. In this work we will show, that the GA can be used to determine the repulsive part of PEC (*e.g.* part above the dissociation limit) based on $bound \rightarrow free$ dispersed fluorescence spectra. Moreover, we show that the GA can also be used to obtain the whole PEC based on dispersed fluorescence spectra encompassing both $bound \rightarrow free$ and $bound \rightarrow bound$ components.

### General idea of the GA

**Figure 1:**
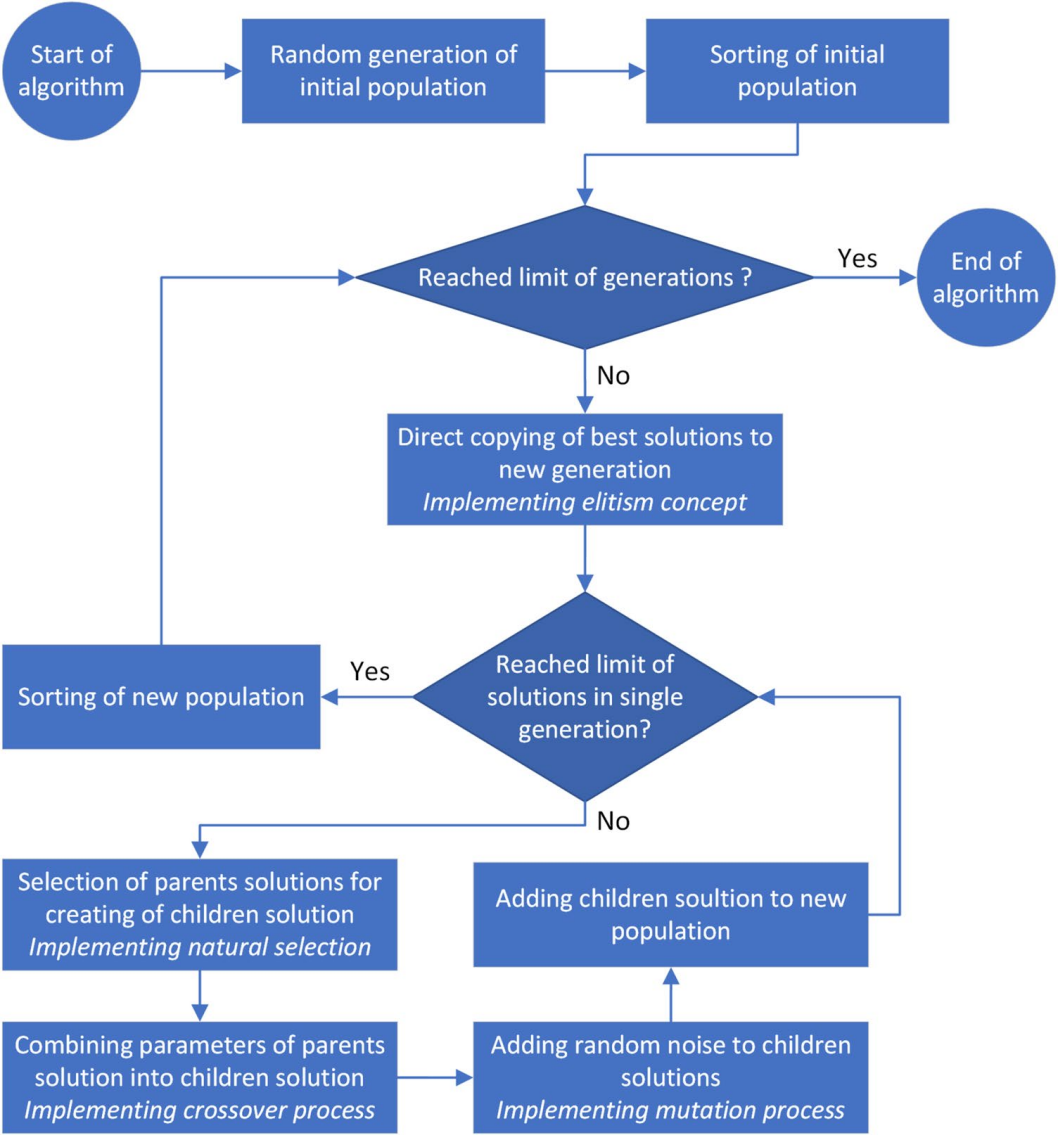
General scheme of the genetic algorithm (GA).

The detailed description of the GA can be found elsewhere^12^, so here only necessary details will be presented. The general scheme of the GA is shown in Fig. 1. The GA is based on the concept of natural selection, which promotes the best individuals in the population. Therefore, in order to be able to apply the GA to solve a given optimization problem, it must be possible to sort a group of potential solutions (so called *candidate solutions*, CS) in order to identify the best ones.

In the first step of the GA, an initial group of CS (so called initial population) is created. Parameters of solutions from this group can be chosen randomly. The number of solutions in the population is determined arbitrarily and depends on the complexity of the problem and usually ranges from several dozen to tens of thousands. Next, the population is sorted, so the best solutions are at the head of list of CS. In the next stages, further populations of solutions are generated iteratively. Generation of new population is terminated *e.g.* after reaching given limit of generations. The process of creation of new population uses mechanisms related to the evolution of living organisms: previously mentioned natural selection and also exchange of genetic material (crossover), and mutations. To create single CS for a new generation from the current population, the two parent solutions are selected. The selection is made randomly, however, to mimic natural selection, the probability of selecting solutions that better solve the considered optimisation problem are higher than the probability of selecting less optimal solutions (it means, that solution in each generation should be sorted and selection algorithm should prefer solutions from the top of sorted list). After selecting the two parents solutions, the new solution is created *e.g.* by averaging the parameters of parents solutions, which mimic the biological crossover process. Next, to imitate the mutation process, small random noise is added to parameters of the children solution. To create the new generation, the process of creation of the children solution is repeated, until the given population size limit is reached. Moreover, to avoid losing the best solutions, few best solutions from the current generation are copied directly (without any changes) to the new generation. It is so-called elitism concept, which does not exist in nature.

### Implementation of the GA to obtain PEC based on dispersed fluorescence spectra

**Figure 2:**
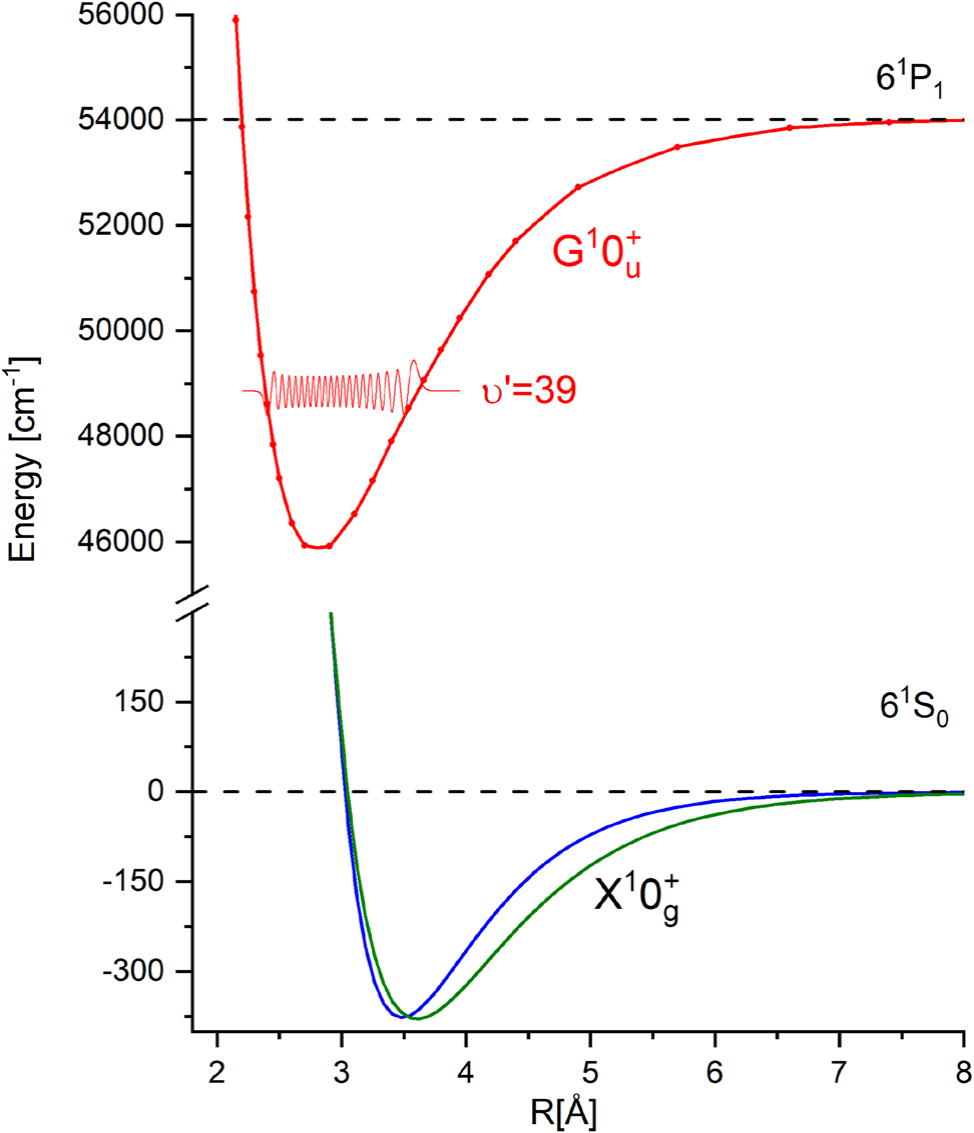
The potentials of states engaged in the analysed transitions. Red line with red points—representation of the $G^10_u^+$ state from Krośnicki et al.^13^. The ground state potential $X^10_g^+$ is depicted by green line (Morse function from Krośnicki et al.^13^) and blue line showing EMO representation from this work. Thin red line depicts the wavefunction of $\upsilon '=39$ level in the $G^10_u^+$ state.

In the case of diatomic van der Waals molecules studied in our laboratory, the dispersed fluorescence spectrum arises as a result of spontaneous deexcitation of molecules excited by a laser beam from a ground electronic state to a selected vibrational level of the excited electronic state. The deexcitation can occur to different electronic states that lie below the excited state, however, *e.g.* due to transition dipole moment (TDM) and distribution of Franck-Condon factors, a strong fluorescence associated with deexcitation to the ground state occurs. Later in the article, we focus on the spontaneous deexcitation from a single vibrational level to the ground molecular state. The shape of the observed spectrum depends on PEC of both states involved in the transition, however, we assumed that PEC of the excited state is known (it can be determined *e.g.* from the LIF excitation spectra). We also assumed, that the PEC of the ground state is described by an analytical function. Therefore, the purpose of the work is to use the GA to find parameters of analytical representation of PEC of the ground state, which lead to the simulation of dispersed fluorescence spectrum with high agreement with the recorded spectrum. The potentials of states engaged in the analysed transitions are presented in Fig. 2.

The dispersed fluorescence spectrum consists of two contributions: part related with the deexcitation of molecules to the repulsive branch of the PEC of ground electronic state (so-called $bound \rightarrow free$ transitions) and part related with the envelope of spectral lines associated with transitions to ground-state vibrational levels ($bound \rightarrow bound$ transitions). The boundary between both types of transitions can be easily determined as it is equal to the absolute energy of the state from which fluorescence occurs. It is worth noting that the $bound \rightarrow bound$ part of dispersed fluorescence spectrum includes transitions to highly excited vibrational levels of the ground state, which are difficult to study using $bound \leftarrow bound$ excitation spectra measured in the LIF spectroscopy. This problem occurs especially in case of molecules produced in the supersonic molecular beam method, in which only the lowest vibrational levels of the ground state are occupied. However, it should also be remembered that the resolution of dispersed fluorescence spectra is usually much worse than in the case of excitation spectra measured by the LIF method. This is due to the fact, that in case of dispersed fluorescence spectra their resolution is determined by the spectral resolution of the spectrometer, while in case of excitation spectra the spectral resolution is determined mainly by the spectral width of the laser. Therefore, the peaks observed in the $bound \rightarrow bound$ part of the dispersed fluorescence spectra often are associated with transitions to several neighbouring vibrational levels of the ground state.

It is also worth to mention that dispersed fluorescence spectra have very interesting feature as it reproduce the shape of vibrational wave function squared. Therefore, it can be used for determination of the vibrational quantum number of the upper-state level, from which the fluorescence occurs. Here, we quote Tellinghuisen et al. in^14^: *“The resulting diffuse spectra have the appearance of reflection spectra, in which the radial probability distribution in the initial vibrational level is mirrored in the spectrum. If that is the case, a count of the peaks in the spectra gives a direct determination of the vibrational numbering in the excited state”.*

**Figure 3:**
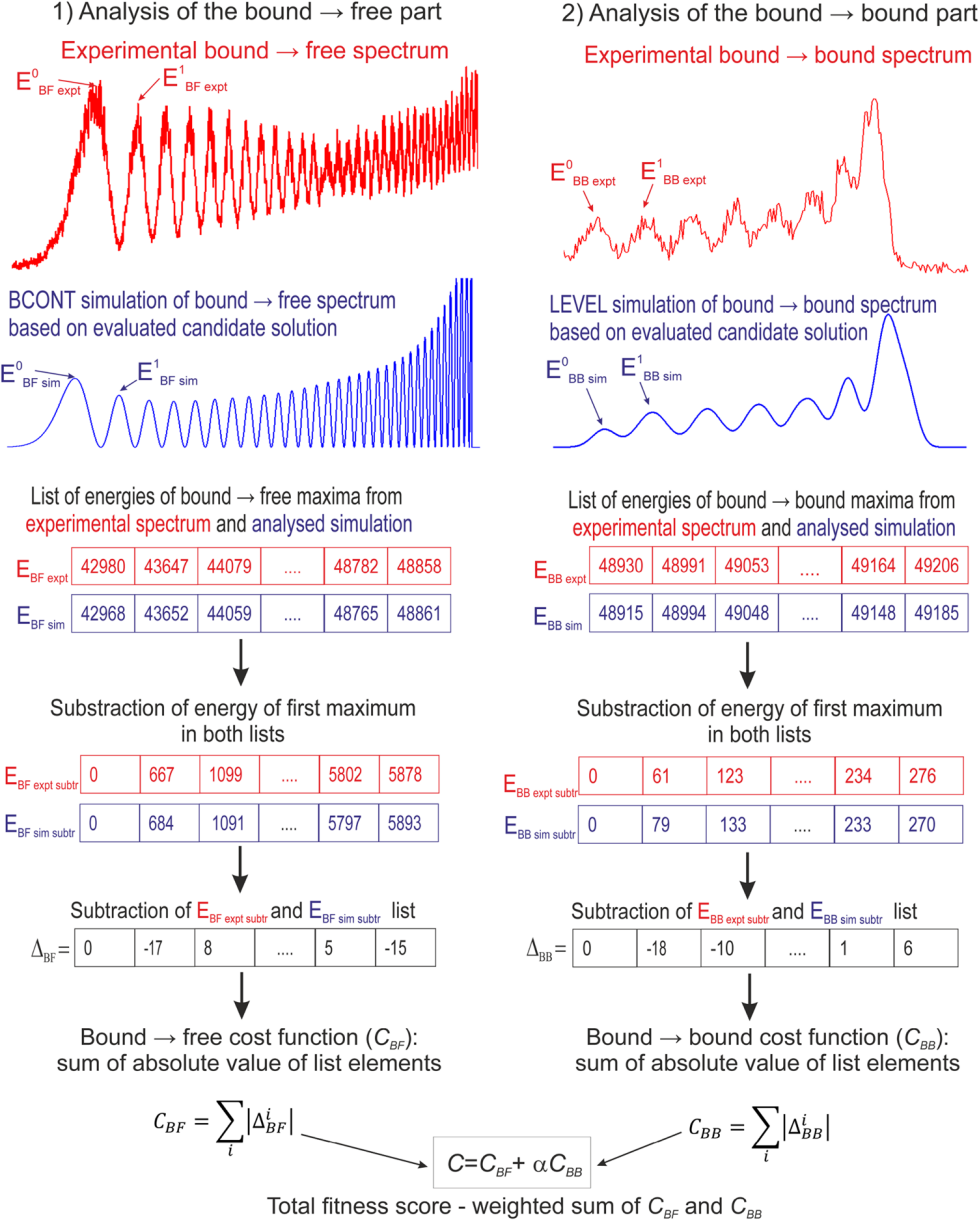
Calculation of fitness score of candidate solution (CS)—a graphical illustration. Details in text.

In our work we assumed, that the PEC of the ground state is represented by four parameters of the expanded Morse oscillator (EMO)^15^ function:

1 $$\begin{aligned} U(r)=D_e[ 1-e^{-\beta (r)(r-R_e)}]^2; \,\,\,\,\,\,\,\,\,\,\,\,\,\,\,\,\,\,\,\,\, \beta (r)=\beta _0+\beta _1\frac{r-R_e}{r+R_e}, \end{aligned}$$

where $D_e$ is the potential well depth, $R_e$ is the equilibrium distance, and $\beta _0$ and $\beta _1$ are parameters that influence width of the potential well. So, a possible solution to our optimisation problem is a set of 4 parameters of EMO potential. It should be also stressed that our choice of the potential is arbitrary, but the algorithm can be easily adopted to obtain parameters of different type of analytical potentials *e.g.* Lennard-Jones, double-exponential-long-range (DELR)^16^ or newly developed so-called modified shifted Morse potential^17^. We also assumed that we were able to determine the ranges in which the optimal values of potential parameters lie. The source of this information may be, for example, *ab-initio* calculations or previous work (*e.g.* parameters of Morse potential obtained using B–S plot method). Our implementation of the GA (the source code is available https://github.com/marek-krosnicki/Diatomic-PEC-Genetic-algohritm ) was created using PYTHON 3 programming language^18^. In the program, in addition to standard PYTHON libraries (*e.g. threading, subprocess, re, os, time*), we used the NumPy^19^ and Matplotlib^20^ libraries. To create the first generation of solutions in the GA, we randomly chose the parameters of each CS from specified ranges (assuming a uniform distribution of probabilities). In the next step, the candidate solutions in the first generation have to be sorted according to quality of given solution. Therefore, one should define a procedure of calculating so-called fitness score, which numerically describes the agreement between simulation of spectrum based on the evaluated CS with the experimental spectrum. The procedure of calculation of the fitness score is presented in Fig. 3.

In the proposed method, the partial fitness score are calculated for $bound \rightarrow free$ and $bound \rightarrow bound$ transitions separately, and finally combined into cumulative fitness score. For both type of transitions, the experimental spectrum contains a series of peaks (maxima), which positions (energies) are collected in two tables $E_{BF\,expt}$ and $E_{BB\,expt}$ (compare with Fig. 3). Next, based on parameters of the evaluated CS, we use BCONT^3^ program to simulate the $bound \rightarrow free$ spectrum, while to simulate the $bound \rightarrow bound$ part of the spectrum we use LEVEL^1^ program. During the GA calculations, in case of discrete energies of $bound\rightarrow bound$ transitions, which are returned by LEVEL program, the Gaussian convolution was used to simulate the broadening introduced by the spectrometer, and obtain the envelope of the $bound\rightarrow bound$ spectrum. Also, we use the standard deviation of the Gaussian distribution $\sigma$=20 cm$^{-1}$, which is comparable to the spectrometer slit-width for experimental spectrum analysed in the article. In order to plot the final simulation, instead of using simple Gaussian convolution we used LEVEL results as an input to PGOPHER program^21^, which can plot more detailed spectrum taking into account not only instrumental broadenings but also rotational temperature. The energies of maxima occurring in both simulations are collected in tables $E_{BF\,sim}$ and $E_{BB\,sim}$. Due to the fact, that in the experimental spectrum the error of determination of absolute energy is significantly larger than the error of determination of relative energy of two maxima for each element in all tables ($E_{BF\,expt}$, $E_{BB\,expt}$, $E_{BF\,sim}$ and $E_{BB\,sim}$), we subtracted the energy of first maximum in given list to obtain new lists of energies: $E_{BF\,expt\,subtr}$, $E_{BB\,expt\,subtr}$, $E_{BF\,sim\,subtr}$ and $E_{BB\,sim\,subtr}$. It should be mentioned, that in case of $E_{BB\,expt}$, the energy of first maximum can be significantly influenced by the energy of last maximum in the $bound \rightarrow free$ spectrum. Therefore, to increase accuracy of computation, one can subtract from $E_{BB\,expt}$ and $E_{BB\,sim}$ the energy of not first but second element of both tables. If the corresponding matrices $E_{BF\,expt\,subtr}$ and $E_{BF\,sim\,subtr}$ or $E_{BB\,expt\,subtr}$ and $E_{BB\,sim\,subtr}$ have different number of elements it means, that the simulation based on the evaluated CS is significantly different from the experimental spectrum. Consequently, we set the fitness score to infinity. In the other case, the elements of matrices $E_{BF\,expt\,subtr} - E_{BF\,sim\,subtr}$ and $E_{BB\,expt\,subtr} - E_{BB\,sim\,subtr}$ were subtracted to obtain two new matrices $\Delta _{BF}$ and $\Delta _{BB}$, respectively, which describe the differences between position of maxima in the experimental spectrum and the simulations for both $bound \rightarrow free$ and $bound \rightarrow bound$ transitions. To compute the partial fitness scores $C_{BF}$ and $C_{BB}$, which describe the agreement of simulations with the experimental spectrum for $bound \rightarrow free$ and $bound \rightarrow bound$ transitions, respectively, the absolute values of elements of the $\Delta _{BF}$ and $\Delta _{BB}$ matrices are added. Finally, to compute the fitness score *C*, which takes into account both $bound \rightarrow free$ and $bound \rightarrow bound$ part of the entire spectrum, the weighted sum of $C_{BF}$ and $C_{BB}$ is calculated:

2 $$\begin{aligned} C=C_{BF}+\alpha C_{BB}, \end{aligned}$$

where $\alpha$ is a weight coefficient. The necessity of using the weighted sum is due to the fact, that $C_{BF}$ and $C_{BB}$ describe the cumulative difference between simulated and observed energies of maxima for $bound \rightarrow free$ and $bound \rightarrow bound$ transitions, respectively. However, in case of the analyzed transitions, the number of maxima in $bound \rightarrow free$ part is significantly higher than in $bound \rightarrow bound$ part, so without using weight, $C_{BF}$ coefficient could dominate *C*. As $\alpha$ we used the ratio of the number of maxima in $bound \rightarrow free$ and $bound \rightarrow bound$ parts of the spectrum.

To implement the crossover process, parameters of the child solution were calculated as weighted average parameters of the parents’ solutions:

3 $$\begin{aligned} X_{child}=\gamma \cdot X_{parent\,A}+(1-\gamma ) \cdot X_{parent\,B}, \end{aligned}$$

where *X* denotes the parameter which is calculated ($D_e$, $R_e$, $\beta _0$ or $\beta _1$), whereas $\gamma$ is a random weight chosen independently for each child solution and each parameter from the range (0,1). The mutation process was implemented by multiplying each parameter of the children solution by a random factor very close to 1:

4 $$\begin{aligned} X_{child\,mut}=X_{child}\cdot (1+\mu ), \end{aligned}$$

where $\mu$ was chosen randomly (normal distribution) from the range (−0.01, 0.01). The elitism concept was implemented by direct copying of 5 best solutions from the previous generation to the new generation. In the proposed implementation of the GA, we also made additional assumption that the limit of the CS in initial generation is twice as large as in subsequent generations. Thanks to this approach, the GA can check more combinations of possible CS in the first step, when parameters are picked out randomly.

It is worth mentioning that instead of analyzing only the energies of the maxima, the spectrum can be also analyzed as a whole, taking into account also the information on the intensities of individual maxima. However, this approach is difficult, because the intensities of individual maxima in the spectrum are influenced not only by the potentials of the states involved in the transition, but also by TDM function. In addition, the distribution of intensities of maxima in the spectrum may be also distorted by different spectrometer sensitivity for different wavelengths. Therefore, in our opinion, it is better to take into account only the energies of maxima, because these depend only on the potentials of the studied electronic states.

## Results

### Tests for artificially generated reference spectra

**Figure 4:**
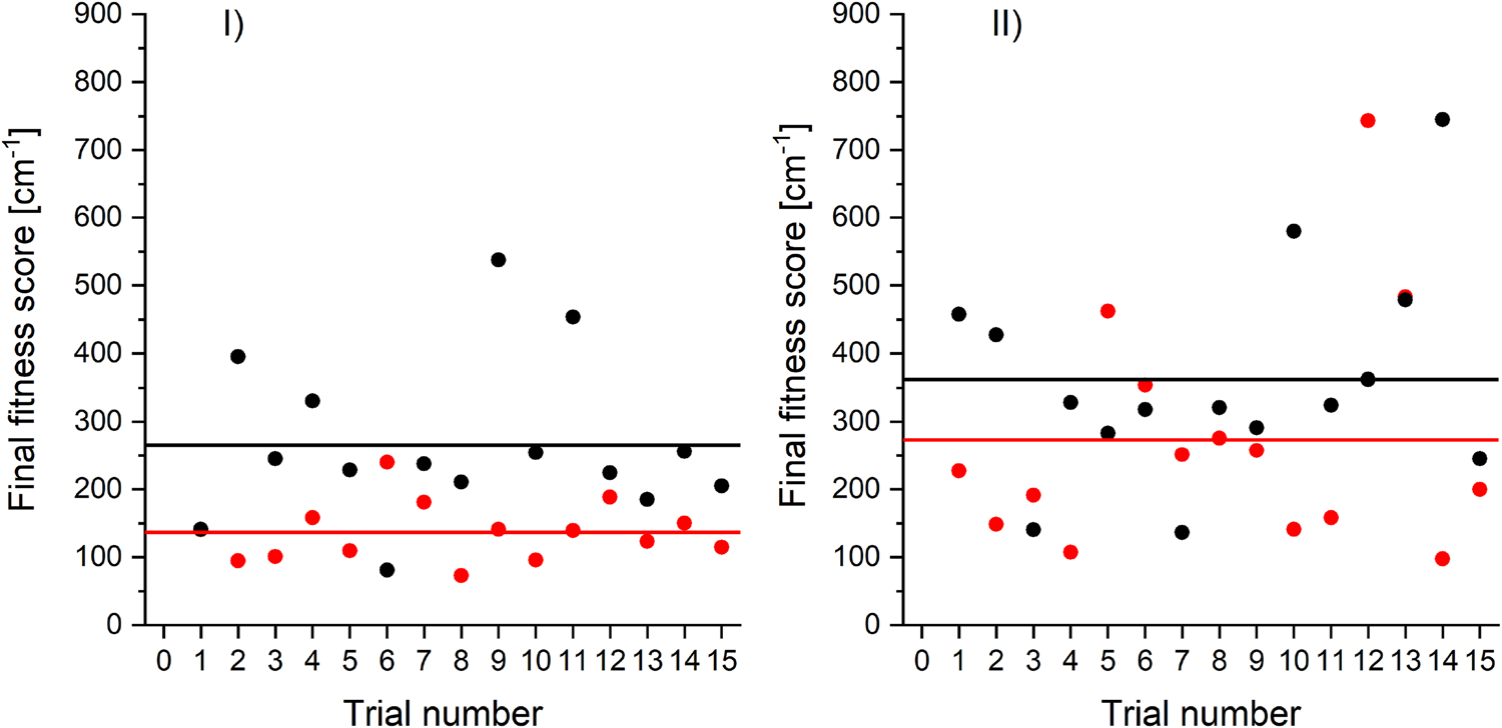
The fitness scores obtained in 15 trials of the GA (red points) and the BF (black points) algorithms. Part I presents results after 10 generations of the GA with 100 CS in each generation (except the initial generation with 200 CS) and the BF with 1200 CS, whereas part II presents the result after 10 generations of the GA with 50 CS in each generation (except the initial generation with 100 CS) and the BF with 600 CS. Averaged fitness score for 15 trials of the GA and the BF are depicted by red and black lines, respectively.

**Figure 5:**
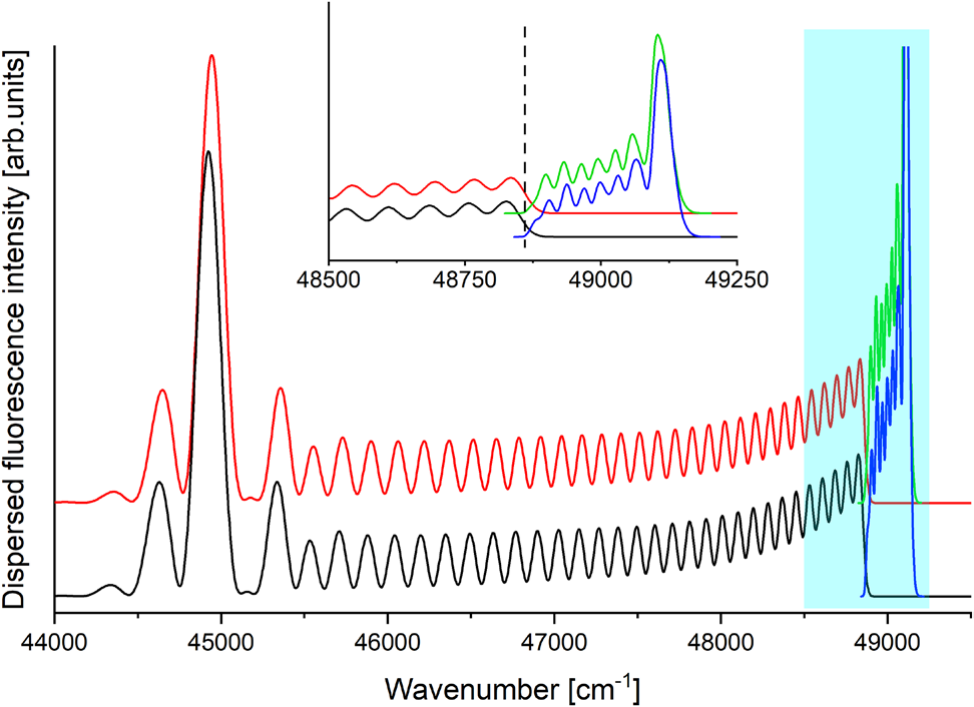
BCONT (red trace) and LEVEL/PGOPHER (green trace) simulations of the reference spectrum (potential reference values—third column of Table 1). BCONT (black trace) and LEVEL/PGOPHER (blue trace) simulations based on potential parameters obtained in one of 15 trials by the GA (GA values—fourth column of Table 1). Inset shows the fragment marked with blue rectangle. The boundary between $bound \rightarrow free$ and $bound \rightarrow bound$ parts of the reference spectrum is indicated by the vertical dashed line.

In order to test the correctness of the proposed algorithm, it was decided to use artificially generated reference spectrum. The test method involves generation of a reference spectrum based on known values of parameters of the PEC and then, using the GA algorithm, to determine these parameters. In other words, the reference spectrum is a simulation based on known values of potential parameters. In the tests it was checked whether GA can reproduce the values of parameters which was used to generate the reference spectrum (see Table 1). It was also tested whether the simulation based on the GA was compatible with the artificially generated reference spectrum (see Fig. 5). The parameters used for construction of the reference spectrum have been selected so that the reference spectrum was similar to the spectrum of the $G0_u^+(\upsilon '=39) \rightarrow X0_g^+$ transition in Hg$_2$^22^. To achieve this, the parameters of the upper $G0_u^+$ state was taken from^13^, whereas the $X0_g^+$ ground state PEC was represented by four-parameter EMO function which parameters was chosen as to be close to the parameters of a Morse function presented in the literature^13^. In Table 1 parameters of EMO potential used to construct reference spectrum are collected as well as searching ranges for each parameter and the exemplary result of the GA with 10 generations and 100 CS in each generation (excluding initial generation which consists of 200 CS). To check the stability, we run the GA 15 times. The obtained fitness scores of the final solutions returned by the GA in each trial are presented by red points in part I of Fig. 4.

**Table 1:** Parameters, searching ranges, reference values and results of the GA obtained for the reference spectrum. As the results of GA, the results of one of 15 trials of GA with fitness score close to the averaged fitness score of all trials (134.7 cm $^{-1}$) were presented. Details in text.

Parameter	Searching range	Reference (expected) value	GA value
$D_e$[cm$^{-1}$]	370–390	379.5	380.3
$R_e$ [Å]	3.5–3.7	3.605	3.621
$\beta _0$[1/Å]	1.1–1.4	1.234	1.204
$\beta _1$ [1/Å]	0–0.1	0.080	0.050

The averaged fitness score for 15 trials is shown with red line. For comparison, we also used the brute force (BF) algorithm to solve the same optimisation problem. The BF generated a set of solutions with randomly chosen parameters and found the solution which led to the simulation with the best agreement with the reference spectrum. This means that the BF algorithm is equivalent to the GA algorithm, which terminates after generating the initial population. The number of solutions picked by the BF algorithm was 1200, the same as the number of solutions evaluated by the GA in the initial generation and subsequent 10 generations. The fitness scores for the results of 15 trials of the BF algorithm and their average are presented in part I of Fig. 4 with black points and black line, respectively. To check the influence of the number of CS in each generation, we redid computation using the GA algorithm with only 50 CS in each generation (excluding initial generation with 100 CS) and the BF algorithm with 600 CS. The results are presented in part II of Fig. 4, where red and black points present fitness scores resulting from the GA and BF algorithms, respectively, while red and black lines in part II indicate averaged fitness score for 15 GA and BF trials, respectively. The averaged fitness score for GA in test with 100 CS in each generation was 137 cm$^{-1}$ with standard deviation (SD) 43 cm$^{-1}$ and for test with 50 CS it was 273 cm$^{-1}$ with SD 175 cm$^{-1}$. For comparison, the averaged fitness score for BF algorithm with 1200 CS was 266 cm$^{-1}$ with SD 119 cm$^{-1}$ and for 600 CS it was 363 cm$^{-1}$ with SD 159 cm$^{-1}$. One can easily see, that regardless of the number of solutions in the population, using the GA algorithm provides (on average) a set of PEC parameters which lead to a simulation with better agreement with the reference spectrum than in case of parameters returned by the BF algorithm with similar computation time. Moreover, using larger number of CS in each generation increases the agreement between simulations obtained using the GA result and the reference spectrum. Fig. 5 presents reference spectrum *i.e.* BCONT and LEVEL/PGOPHER simulations based on EMO potential of the ground state with reference values from third column of Table 1 compared with simulations based on parameters of EMO potential of the ground state returned by one of 15 GA trials (compare with fourth column of Table 1) with fitness score close to the average value depicted by red line in part I of Fig. 4). One can see, that the agreement between simulation based on the GA result and the reference spectrum is very high. Also, the fitness score, which measures the cumulative discrepancy between simulated energies of maxima and the energies observed in reference spectrum, is small. The score is equal to 134.7 cm$^{-1}$ so it means, that - on average - the discrepancy between energy of maximum in simulation and in reference spectrum is less than 3.5 cm$^{-1}$.

**Figure 6:**
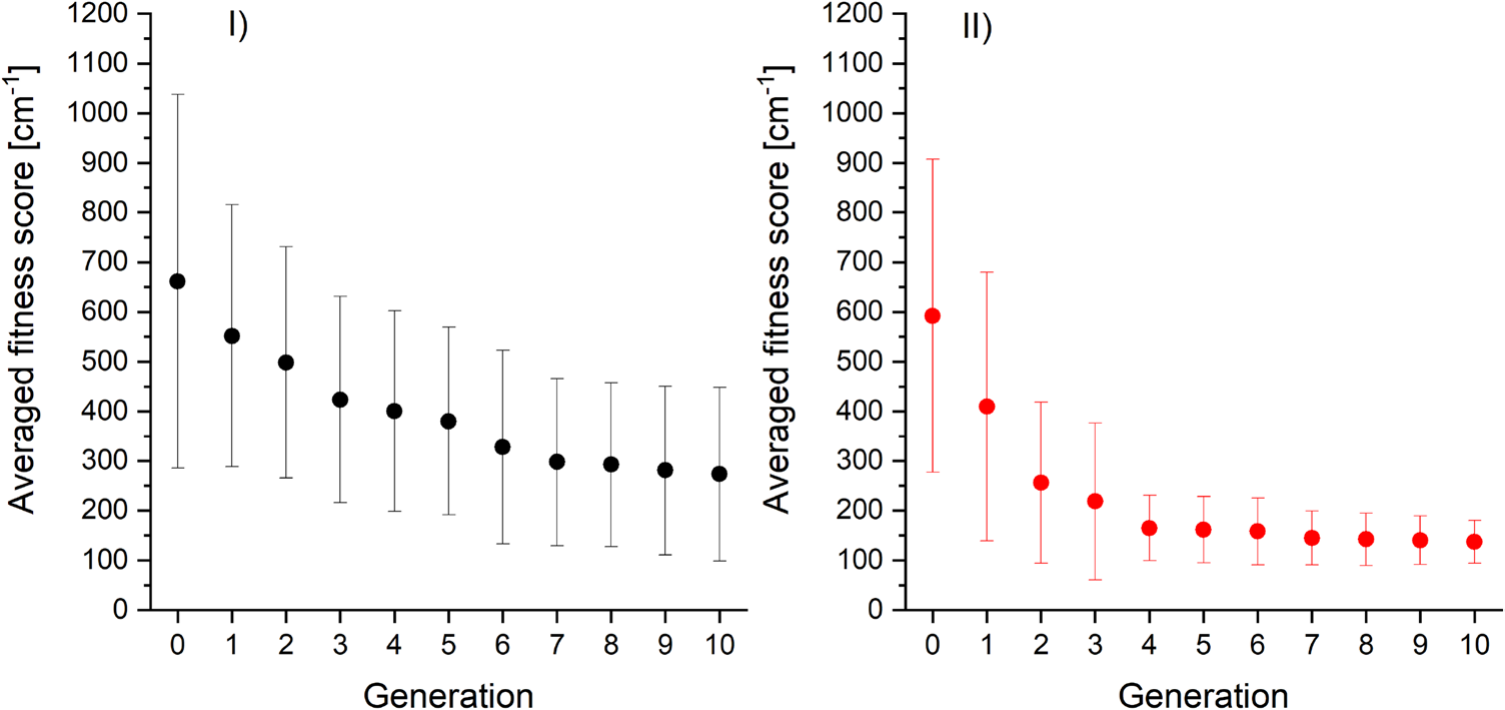
Best fitness score averaged over 15 trials obtained for subsequent generations of the GA for 50 CS (part I) and 100 CS (part II) in each generation. Details in text.

Figure 6 presents the fitness score after each generation averaged over 15 trials of the GA with 100 (part I, red points) and 50 (part II, black points) CS in each generation. The error bars depict the sample standard deviation (SSD). Fig. 6 shows that both for 100 and 50 CS in generation, there is a successive decrease of the averaged fitness score for subsequent generation. Moreover, for the GA with 100 CS in each generation, the obtained SSDs in each generation are significantly smaller than those in case of the GA with 100 CS in a generation. Tests conducted using computer with Intel(R) Xeon(TM) E3-1240 v3 processor with 32 GB RAM showed that the execution time of the GA algorithm with 10 generations and 100 CS in each generation is about 20 minutes, whereas using 50 CS in each generation reduce the execution time to 10 minutes.

### Test with experimental spectrum of the $G0_u^+(\upsilon '=39) \rightarrow X0_g^+$ transition in Hg$_2$

**Figure 7:**
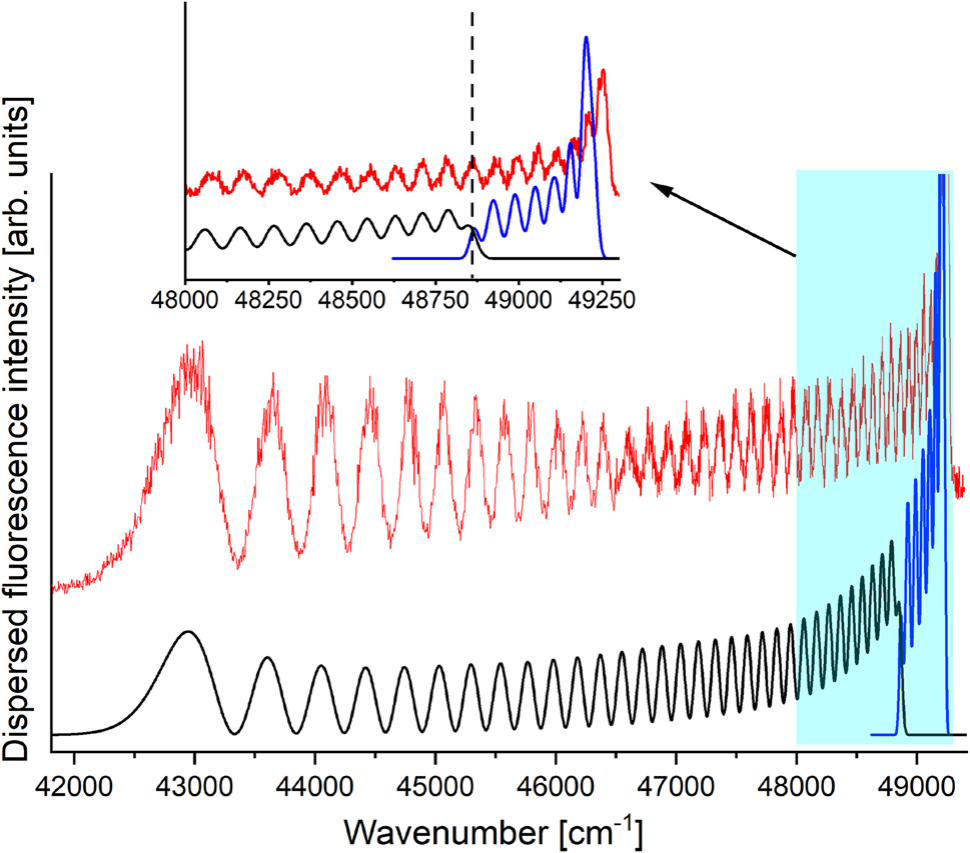
Experimental spectrum of the $G0_u^+(\upsilon '=39) \rightarrow X0_g^+$ transition in Hg$_2$ (red line) and its simulation based on result of the GA (black and blue lines for $bound \rightarrow free$ and $bound \rightarrow bound$ transitions, respectively). The experimental spectrum was recorded with 20 cm$^{-1}$ spectrometer slit-width. The same Gaussian broadening was applied to $bound\rightarrow bound$ transitions during execution of the GA and as a parameter in PGOPHER simulation (blue line). Inset shows the fragment marked by blue rectangle. The boundary between $bound \rightarrow free$ and $bound \rightarrow bound$ parts of the spectrum is depicted by vertical dashed line. Details in text.

The proposed GA was also tested on real experimental spectrum of the $G0_u^+(\upsilon '=39) \rightarrow X0_g^+$ transition in Hg_2_^22^. To determine the parameters of EMO potential with 4 parameters representing the $X0_g^+$ state, we run the GA with 10 generations and 150 CS in each generation (excluding initial generation with 300 CS). The searching ranges and parameters obtained by the GA are presented in Table 2. These ranges were determined on the basis of results of *ab-initio* calculations and previous experimental works^13,23,24^. The experimental spectrum and its simulation based on the result of the GA are presented in Fig. 7. The agreement between recorded spectrum and its simulation based on the GA result is high. The significant discrepancy occurs only for the most extreme maximum on the right-hand side of the spectrum. However, its correct simulation would require the potential well of the ground $X0_g^+$ state to be much deeper, which contradicts the results of other studies to date an also numerous ab-initio results (e.g.^23,24^). The reason for the discrepancy is probably that, according to the information provided by the co-author of the experimental measurements, the spectrometer detector could have been oversaturated in the problematic part of the spectrum, which resulted with the fact that the strongest maximum could be split (i.e., in the place of the actual maximum, a minimum appeared due to oversaturation). It is also worth to explain the reason for the discrepancy between the equilibrium distance $R_e$ determined by GA and the $R_e$ from the work^13^. The repulsive parts of PECs are similar (compare Fig. 2). In order to precisely determine the shape of the well, one needs the measurement of *bound* $\leftarrow$ *bound* excitation spectra originated from a set of excited vibrational levels of the ground electronic state and these levels are not accessible in supersonic beam experiment.

**Table 2:** Parameters of EMO potential, searching ranges and the GA values obtained using experimental spectrum of the $G0_u^+(\upsilon ^{\prime }=39) \rightarrow X0_g^+$ transition in Hg$_2$^22^. Values of parameters of a Morse representation of Krośnicki et al.^13^ are collected for comparison. Details in text.

Parameter	Searching range	GA value	Value of Ref.^13^
$D_e$[cm$^{-1}$]	370–390	376.9	379.5
$R_e$[Å]	3.4–3.7	3.483	3.605
$\beta _0$ [1/Å]	1.1–1.7	1.515	N.A.
$\beta _1$ [1/Å]	0–0.1	0.025	N.A.

## Conclusions

We proposed a method based on the Genetic Algorithm (GA) approach, which can by used to obtain the parameters of an analytical potential energy curve (PEC) which is valid both below and above the dissociation limit. The parameters of PEC are obtained based on the experimental dispersed flourescence spectrum, which contains information about both $bound\rightarrow bound$ and $bound \rightarrow free$ transitions. The GA was tested on obtaining parameters of an expanded Morse oscillator (EMO) potential, but it can be easily modified to work with different types of analytical potentials. In our implementation of the GA, to simulate the spectrum we used well established BCONT and LEVEL programs, however, they are relatively slow. Due to the fact that these programs are used repeatedly during the operation of the GA, they have very large impact on the GA execution time. The GA would work much faster if it employs a procedure that uses the capabilities of modern GPUs to solve the appropriate Schrödinger equations.

The tests show very good accuracy of simulation based on GA results with artificially generated reference spectrum as well as with the experimental one. However, it is also worth analyzing the limitations in the accuracy of the proposed method. In the case of simulation of the experimental spectra, deviations of the simulation from the experimental spectrum (and thus a high value of fitness score) may be associated with the fact that the selected analytical function may not be suitable for the correct representation of the actual potential of the lower state. What is more, the simulated energies are influenced not only by the potential of the lower state but also by the potential of the upper state. In the method, we assumed that the second potential is known, but its imperfections can affect the quality of the simulation. In the case of simulation of the reference spectra, it is possible to numerically increase the accuracy (i.r., obtaining lower fitness scores) by increasing the number of solutions in individual populations. However, it should be remembered that this will not contribute to obtaining a simulation that—in a visual assessment—would be more accurate. This is due to the fact that in the case of $bound\rightarrow free$ spectra, the observed structures are not narrow lines (as in the case of $bound\leftarrow bound$ in LIF excitation spectra), but relatively wide peaks, because they reflect the shape of the wavefunction squared of the emitting vibrational level in the upper state. For example, in the case of the analyzed experimental spectrum, the full width at half maximum (FWHM) of individual peaks varied from about 250 cm$^{-1}$ in the left-hand part of the spectrum to about 30 cm$^{-1}$ near the dissociation limit. Another, very important limitation of the proposed method concerns the scope of the searching ranges. If any of the true (globally optimal) potential parameters lies outside its searching range, then the GA will return a solution that is optimal locally (within the analyzed searching ranges) and not globally. A clue of that such a situation may occur (which, however, does not have to occur in every case) is that one of the parameters returned by the GA lies very close to the end of its searching range.

## Data Availability

The source code of algorithm generated during the current study is available in the public repository: https://github.com/marek-krosnicki/Diatomic-PEC-Genetic-algohritm.
